# High Prevalence of *bla*_*CTXM*–1_/IncI1-Iγ/ST3 Plasmids in Extended-Spectrum β-Lactamase-Producing *Escherichia coli* Isolates Collected From Domestic Animals in Guadeloupe (French West Indies)

**DOI:** 10.3389/fmicb.2022.882422

**Published:** 2022-05-16

**Authors:** Gaëlle Gruel, David Couvin, Stéphanie Guyomard-Rabenirina, Guillaume Arlet, Jean-Christophe Bambou, Matthieu Pot, Xavier Roy, Antoine Talarmin, Benoit Tressieres, Séverine Ferdinand, Sébastien Breurec

**Affiliations:** ^1^Transmission, Reservoir and Diversity of Pathogens Unit, Pasteur Institute of Guadeloupe, Pointe-à-Pitre, France; ^2^U1135-Cimi-Paris, Sorbonne University, Paris, France; ^3^INRAE, ASSET, Petit-Bourg, France; ^4^Veterinary Clinic, Baie-Mahault, France; ^5^INSERM 1424, Center for Clinical Investigation, University Hospital Center of Guadeloupe, Pointe-à-Pitre, France; ^6^Faculty of Medicine Hyacinthe Bastaraud, University of the Antilles, Pointe-à-Pitre, France

**Keywords:** Enterobacteriaceae, *Escherichia coli*, ESBL, pets, shelter, nanopore, plasmid

## Abstract

Extended-spectrum β-lactamase-producing Enterobacteriaceae (ESBL-E) have been classified in the group of resistant bacteria of highest priority. We determined the prevalence of ESBL-E collected in feces from household and shelter pets in Guadeloupe (French West Indies). A single rectal swab was taken from 125 dogs and 60 cats between June and September 2019. The prevalence of fecal carriage of ESBL-E was 7.6% (14/185, 95% CI: 4.2-12.4), within the range observed worldwide. The only risk factor associated with a higher prevalence of ESBL-E rectal carriage was a stay in a shelter, suggesting that refuges could be hotspots for their acquisition. All but one (*Klebsiella pneumoniae* from a cat) were *Escherichia coli*. We noted the presence of a *bla*_*CTX–M*–1_/IncI1-Iγ/sequence type (ST3) plasmid in 11 ESBL-producing *E. coli* isolates belonging to ST328 (*n* = 6), ST155 (*n* = 4) and ST953 (*n* = 1). A *bla*_*CTX–M*–15_ gene was identified in the three remaining ESBL-E isolates. The *bla*_*CTX–M*–1_ and most of the antimicrobial resistance genes were present in a well-conserved large conjugative IncI1-Iγ/ST3 plasmid characterized by two accessory regions containing antibiotic resistance genes. The plasmid has been detected worldwide in *E. coli* isolates from humans and several animal species, such as food-producing animals, wild birds and pets, and from the environment. This study shows the potential role of pets as a reservoir of antimicrobial-resistant bacteria or genes for humans and underlines the importance of basic hygiene measures by owners of companion animals.

## Introduction

Currently, resistance to antibiotics is one of the most urgent public health threats in the world and could be responsible for more deaths than cancer by 2050 (i.e., 10 million deaths per year) (O’Neill, 2016). This complex problem involves humans, animals, and the environment. Recently, the World Health Organization published a global priority list of antibiotic-resistant bacteria, on which third-generation cephalosporin (3GC)-resistant Enterobacteriaceae were in priority 1 group ((World Health Organization, 2020). This type of resistance is mediated mainly by acquired extended-spectrum β-lactamase (ESBL) genes located on plasmids (Bush and Bradford, 2020). ESBL enzymes can confer resistance to all β-lactams except for carbapenems and cephamycins and are frequently associated with genes that confer resistance to a wide variety of antimicrobial agents.

During the past 30 years, the number of companion animals in developed countries has been increasing. For example, in 2020, approximately 45% of households in the United States had one or more dogs and 26% had one or more cats, the highest rates since measurement began in 1982 (AMVM, 2020; AMVM, 2021). In the same year, the estimated number of households in Europe that owned at least one companion animal was about 88 million, most of which were dogs and cats, representing 89.8 and 110.1 million animals, respectively (The European Pet Food Industry, 2021). To our knowledge, no data on dog and cat populations in Guadeloupe are available.

Since the first report of an ESBL-producing uropathogenic *Escherichia coli* isolate from a dog in Spain in 1998 (Teshager et al., 2000), more reports of ESBL-producing Enterobacteriaceae (ESBL-E) in animals have been published globally (Bush and Bradford, 2020; Salgado-Caxito et al., 2021), raising concern that animals are possible sources and reservoirs of ESBLs for humans (Dupouy et al., 2019). Close contact between pets and owners provides opportunities for transmission of antimicrobial-resistant bacteria. Feeding with raw pet food was found as risk factor for ESBL-E colonization in cats (Baede et al., 2017).

Guadeloupe, a French overseas territory located in the Caribbean, was considered a very high-resource country in 2013 (Human Development Report, 2013). Data on resistance to antibiotics in Guadeloupe are scarce and recent. The University Hospital of Guadeloupe faced the emergence of carbapenemase-producing Enterobacteriaceae isolates and a very high incidence of nosocomial ESBL-E infections (Bastian et al., 2015; Breurec et al., 2015; Le Terrier et al., 2021). A moderate prevalence of ESBL-E (around 5%) was observed in studies on community human urinary tract infections (Guyomard-Rabenirina et al., 2016), on fecal carriage by domestic animals and livestock (Gruel et al., 2021) and on wild fauna (Guyomard-Rabenirina et al., 2020). Guadeloupe is an ideal place to study the circulation of multi-resistant bacteria and resistance genes because of its insularity and small population (395,700 inhabitants) and area (1,436 km^2^). The primary objective of our study was to determine the prevalence of ESBL-E in feces from household and shelter pets in Guadeloupe. The secondary objectives were to determine the risk factors associated with ESBL-E colonization in pets and to use whole-genome sequencing (WGS) to identify the genetic background of ESBL-E collected from pets and the genetic basis for resistance to 3GC.

## Materials and Methods

### Sampling

Between June 2019 and September 2019, single rectal swabs were taken from 125 dogs and 60 cats. Of the 185 pets investigated, 15 were from the main animal shelter of Guadeloupe and 170 from seven veterinary clinics that provide preventive health services, vaccination or medical consultation in 18 cities throughout Guadeloupe and Les Saintes, a small nearby island. None of the animals displayed clinical signs of diarrhea and had received antibiotic treatment in the 3 months before inclusion.

For each animal examined, a questionnaire eliciting information on factors such as age, municipality of residence, general health and lifestyle (indoor or free roaming outdoors, close contact with other animals or not) was completed by each veterinarian based on the owners’ declarations. Samples were stored in the veterinary clinics at + 4°C until shipment to the microbiology laboratory of the Pasteur Institute of Guadeloupe (within 48 h), where they were immediately analyzed. The project was approved by the Committee for Ethics in animal experiments of the French West Indies and Guyana (reference HC_2020_1). The animals were cared for and used according to French decree No. 2013-118 of 1 February 2013 on the protection of animals, which meets European Union Directive 2010/63 on the protection of animals used for experimental and other scientific purposes.

### Enterobacteriaceae: Isolation, Identification and Antibiotic Susceptibility Testing

The bacterial populations of interest were enriched and preselected by incubating each rectal swab specimen in 9 mL of Luria-Bertani (LB) broth for 16–20 h at + 37°C; of this, 100 μL were inoculated on CHROMagar™ CCA supplemented with ceftriaxone (4 mg/L) (Guyomard-Rabenirina et al., 2020). Presumptive Enterobacteriaceae colonies were identified on selective media on morphological criteria (blue, smooth, round colonies were presumed to be *E. coli*, and pink, smooth, round colonies were presumed to be members of *Klebsiella, Enterobacter*, or *Serratia* spp. (KES group), isolated randomly and identified in the Api 20E test (BioMérieux, Marcy-l’Etoile, France). Three colonies were randomly identified for each identical morphology.

The susceptibility of the Enterobacteriaceae isolates to antibiotics was assessed with the disk diffusion method on Mueller-Hinton agar according to the 2020 guidelines of CA-SFM/EUCAST (CA-SFM/EUCAST, 2020). The antimicrobial agents tested were ampicillin 10 μg, amoxicillin/clavulanic acid 20/10 μg, ticarcillin 75 μg, cefoxitin 30 μg, cefotaxime 5 μg, ceftazidime 10 μg, cefepim 30 μg, temocillin 30 μg, ertapenem 10 μg, nalidixic acid 30 μg, ciprofloxacin 5 μg, fosfomycin 200 μg, amikacin 30 μg, gentamicin 10 μg, tetracycline 30 μg, tigecycline 15 μg and trimethoprime/sulfamethoxazole 1.25/23.75 μg. Growth inhibition diameters were measured in the automated Adagio system (Bio-Rad). *E. coli* ATCC 25922 and *K. pneumoniae* ATCC 700603 were used as control strains. Isolates with a resistant or intermediate phenotype were classified together for the analysis. If more than one Enterobacteriaceae isolate within the same species with the same antibiotic susceptibility pattern was isolated from the same animal, only one randomly chosen isolate was analyzed. A double-disk synergy test was performed for all isolates (Breurec et al., 2013).

### Core Genome Phylogenetic Analyses and Antibiotic Resistance Genes

WGS was performed on 14 ESBL-E isolates (13 *E. coli* and 1 *K. pneumoniae*) collected from the pets included in this study at the “Plateforme de microbiologie mutualisée” of the Pasteur International Bioresources network (Institut Pasteur, Paris, France). DNA was extracted with a DNA minikit (Qiagen, Hilden, Germany). The libraries were prepared with a Nextera XT kit (Illumina), and sequencing was performed with the NextSeq 500 system (Illumina), generating 150 bp paired-end reads, for a mean 48-fold coverage. The reads were deposited in the NCBI-SRA public archives under the project’s accession number PRJNA798606. They were trimmed and filtered with AlienTrimmer (Criscuolo and Brisse, 2014).

Genomes were assembled with SPAdes software v3.9.0 (Bankevich et al., 2012). The quality of the assemblies was checked with QUAST software (Gurevich et al., 2013), giving a mean N50 of 112,604 (minimum, 61,542; maximum, 134,844). For ESBL-producing *E. coli* (ESBL-Ec) isolates, a core genome was extracted with Roary software (Page et al., 2015). Recombination sequences were identified and removed from the global core genome alignment with ClonalFrameML software (Didelot and Wilson, 2015), giving a global alignment of 3,367,757 bp, 3,429 genes shared by 100% of the bacterial isolates, and 1,254 polymorphic sites. Maximum likelihood (ML) phylogenetic reconstruction was performed with RAxML version 8.2.8 (Stamatakis, 2014), the GTR-CAT model, and 100 bootstrap replicates, and the tree was drawn with iTOL (Letunic and Bork, 2021). *In silico* multilocus sequence typing (MLST) was done with MLST software^1^ against the PubMLST database (Jolley et al., 2018). *E. coli* was phylotyped with the EzClermont web application (Waters et al., 2020). ABRicate^2^ was also used with the default parameter to assess the content of plasmids and antibiotic resistance genes associated with PlasmidFinder and ResFinder databases, respectively (Zankari et al., 2012; Carattoli et al., 2014).

### *In silico* Detection of Plasmid and Syntenic Analysis

To investigate the presence of an IncI1-Iγ/ST3 signature plasmid in *E. coli* susceptible to 3GC (3GCS-Ec), 51 3GCS-Ec were isolated from frozen stools collected from 51 randomly chosen pets included in this study. A previously described PCR method was used to screen for isolates carrying an IncI1 plasmid (Carattoli et al., 2005). Four IncI1-Iγ positive *E. coli* isolates were identified. WGS was performed as described above. An IncI1-Iγ/ST3 signature was recovered in one isolate (pEC345).

We performed nanopore sequencing (Oxford Nanopore Technology^®^^3^) to fully sequence IncI1-Iγ/ST3 plasmids in two ESBL-Ec isolates representative of the genomic diversity of our set of ESBL-Ec, namely EC405 and EC440, and on 3GCS-Ec, namely pEC345, as previously described (accession numbers pEC405: CP094200; pEC440: CP094199; pEC345: CP094198) (Guyomard-Rabenirina et al., 2020). MinION reads were base-called with Guppy software v3.6.0 and further de-barcoded and screened for quality (resulting in mean q > 11) in EPI2ME v2020.2.10 “Fastq Barcoding workflow.” A hybrid assembly was then performed from both high-quality Illumina and nanopore reads through the Unicycler pipeline (Wick et al., 2017). PlasmidFinder and pMLST were used to identify plasmids with an IncI1/ST3 signature. Antibiotic resistance was further confirmed by ResFinder. The *bla*_*CTX–M*–1_/IncI1/ST3 profile has been described in three previously assembled and annotated plasmids carried by *E. coli* from Guadeloupe, pEC38 (GenBank accession number CP053677) associated with a bloodstream infection in a patient living in Guadeloupe, pEC1 from a bird (CP053560) and pEc7 (CP053679) from a rat (Guyomard-Rabenirina et al., 2020). Open reading frames and pseudogenes were predicted by RAST 2.0 (Brettin et al., 2015) and were further annotated in BLASTP/BLASTN against the UniProtKB/Swiss-Prot (Boutet et al., 2016). Mobile elements and resistance genes were annotated with ISfinder (Siguier et al., 2012), ResFinder (Zankari et al., 2012) and PlasmidFinder (Carattoli et al., 2014) in online databases. Multiple and paired sequences were compared with MAUVE (Darling et al., 2004) and Easyfig (Sullivan et al., 2011). The nucleotide sequence of pEC405 was searched with Mash Screen (Ondov et al., 2019) in the PLSDB database (a resource containing 34,513 plasmid sequences collected from the NCBI nucleotide database) (Galata et al., 2019).

### Conjugation Efficiency of *bla*_*CTXM*–1_/IncI1-Iγ/ST3 Plasmids

Conjugation in LB was performed with *E. coli* K12 J5 resistant to sodium azide as the recipient strain, as described previously (Wang et al., 2008). We used the 4 *bla*_*CTX–M*–1_ positive *E. coli* isolates (pEC38, pEC405, pEC440, pEC1, and pEC7) described above as plasmid donors. The donor and recipient isolates were grown separately in 10 mL of fresh LB broth during 6 h at 37°C with shaking. Optical densities were measured at 600 nm to ensure that cultures of donor and recipient were in logarithmic phase. The donor and recipient strains were mixed in equal volume (1 mL) in 8 mL of fresh LB broth and incubated overnight with shaking. Transconjugants were selected on Drigalski agar (Bio-Rad) supplemented with sodium azide (500 mg/L; Sigma Chemical Co., United States) and cefotaxime (4 mg/L; Sigma Chemical Co., United States). Conjugation experiments were performed at a donor/recipient ratio of 2/1. All conjugation experiments were performed in triplicate. Each donor strain and recipient isolate were sub-cultured on media with cefotaxime and/or sodium azide as controls. After 24 h, the plates were counted, and five colonies were selected from each agar plate for verification by PCR of the presence of an IncI1 plasmid, as described above. The approximate conjugation efficiency was determined as the ratio of the number of transconjugants obtained to the number of donor cells.

### Statistical Analysis

Quantitative variables were summarized as medians with interquartile ranges (IQRs), and categorical data were expressed as percentages. In bivariate analyses, the χ^2^ test (or Fisher’s exact test when appropriate) was used to compare categorical variables. A logistic regression model was used to identify factors associated with the presence of ESBL-E in cats and dogs and to calculate crude and adjusted odds ratios and their 95% confidence intervals (95% CI). Multivariate analyses were not performed due to the low number of cases of ESBL-E in cats (6 cases) and in dogs (8 cases). We considered *P* < 0.05 to be significant. Microsoft Access 2003 was used for data entry. Statistical analyses were performed with Stata Version 10 and SPSS (V21, IBM SPSS Statistics, Chicago, IL, United States).

## Results

### Prevalence and Risk Factors of ESBL-E

During the first sampling campaign, between June and September 2019, a total of 185 pets (80 males, 105 females; median age, 18 months [IQR (7–48)] were included. The prevalence of fecal carriage of ESBL-E was 7.6% (14/185, 95% CI: 4.2–12.4): 6 cats (10.0%) and 8 dogs (6.4%) (Table 1). No simultaneous carriage of ESBL-E isolates belonging to different species was detected. A total of 14 ESBL-E were recovered, all but one of which (*K. pneumoniae* from a cat) were *E. coli*. They were resistant to the β-lactams tested, except for cefoxitin (0% resistance), ertapenem (0% resistance), and temocillin (7% resistance). In addition, they had high rates of resistance to tetracycline (86%) and a high rate of susceptibility to trimethoprime/sulfamethoxazole (79%), nalidixic acid (86%), ciprofloxacin (86%), fosfomycin (93%), tigecycline (100%), amikacin (100%), and gentamicin (100%). The double-disk synergy test was positive for all isolates. If we included only pets from veterinary clinics, the prevalence of ESBL-E was 4.7% (95% CI: 2.1–9.1). Most of them were seen for vaccination (*n* = 73, 42.9%), surgery (*n* = 31, 18.2%), preventive health visit (*n* = 26, 15.3%) or skin and soft tissue infection (*n* = 12, 7.1%).

**TABLE 1:** Risk factors for extended-spectrum beta-lactamase producing-*Enterobacteriaceae* rectal carriage in cats (*N* = 60) and dogs (*N* = 125) from Guadeloupe (French West Indies).

	Cats (*N* = 60)						Dogs (*N* = 125)					
	Cats	ESBL-E*^a^* carriage	%	p	OR	95% CI	Dogs	ESBL-E carriage	%	p	OR	95% CI
Sex												
Female	27	1	3.7	0.209	1		75	3	3.8	0.151	1	
Male	33	5	15.2		4.7.	0.5–41.7	47	5	10.6		3.0	0.7–13.2
Age ≥ 20 months												
No	37	6	16.2	0.075	1		55	1	1.8	0.070	1	
Yes	22	0	0.0		nc*^b^*		66	7	10.6		6.4	0.8–53.8
Shelter stay												
No	51	1	2.0	< 0.001	1		119	7	5.9	0.333	1	
Yes	9	5	55.6		62.5	5.8–672.8	6	1	16.7		3.2	0.3–31.2
Chronic disease												
No	55	6	10.9	1.000	1		118	5	4.2	0.005	1	
Yes	5	0	0.0		nc		7	3	42.9		17.0	3.0–97.0
Antibiotic administration ≤ 3 months												
No	52	6	11.5	0.585	1		106	6	5.7	0.350	1	
Yes	8	0	0.0		nc		19	2	10.5		2.0	0.4–10.5

*^a^Extended-spectrum β-lactamase Enterobacteriaceae. ^b^nc, not calculable.*

A stay in the shelter for cats (OR = 62.5, 95% CI: 5.8–672.8, *p* < 0.001) and chronical disease for dogs (OR = 12.1, 95% CI, 2.0–72.5, *p* = 0.005) were significantly associated with ESBL-E fecal carriage (Table 1).

### Genetic Background and Antimicrobial Resistance Genes of ESBL-Enterobacteriaceae Isolates

**FIGURE 1:**
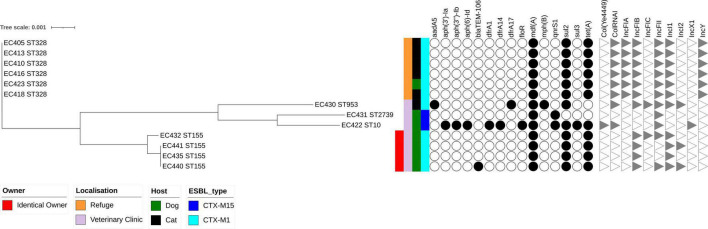
Maximum likelihood phylogenetic tree of 13 extended-spectrum β-lactamase-producing *Escherichia coli* isolates collected in Guadeloupe based on multiple sequence alignments of the 3,429 core genome loci. Sequence type (ST) is indicated for each isolate. Hosts, ESBL genes, site of inclusion, and an identical owner are indicated by vertical colored strips. Other antibiotic resistance genes characterized by ResFinder and Inc. incompatibility group characterized by PlasmidFinder are indicated by black dots and gray triangles, respectively.

All the ESBL-Ec isolates belonged to phylogenetic groups associated with commensalism, including A (*n* = 2, 15%) and B1 (*n* = 11, 85%). Separation into three branches was observed on the phylogenetic tree based on the 13 ESBL-*E. coli* isolates and constructed with RAxML (Figure 1). The first branch consisted only of ST328 isolates (*n* = 6) isolated from five cats and one dog collected at the animal shelter. They were nearly identical at core-genome level, with sequences separated by only 0–5 single nucleotide polymorphisms (SNPs) (Supplementary Table 1). A second branch contained two isolates (EC431 and EC422) assigned to ST10 and ST2739, respectively, from dogs. The last branch consisted of four ST155 isolates collected from dogs. We found 0–1 SNP among three closely related isolates (EC435, EC440, and EC441) belonging to the same owner, whereas 70 SNPs separated them from isolate EC432. All the dogs had attended the same veterinary clinic. The ESBL-producing *K. pneumoniae* was assigned to ST17.

Two types of ESBL gene were identified, *bla*_*CTX–M*–15_ (3/14) and *bla*_*CTX–M*–1_ (11/14) (Figure 1 and Supplementary Table 2). All 11 isolates carrying *bla*_*CTX–M*–1_ were assigned to *E. coli* spp. and belonged to three STs: ST328 (*n* = 6), ST155 (*n* = 4), and ST953 (*n* = 1). The *bla*_*CTX–M*–1_ gene, systematically associated with *sul2* gene, conferring resistance to sulfonamides. An IncI1-Iγ/ST3 signature was detected in all *E. coli* isolates carrying an *bla*_*CTX–M*–1_ gene.

One *bla*_*CTX–M*–15_ was associated with a *K. pneumoniae* isolate (ST17) collected from a cat. It also harbored two genes coding for β-lactamases, *bla*_*SHV*–187_ and *bla*_*TEM*–1*B*_, and also genes coding for *aph*(*3*″)-*Ib, aph*(*6*)-*Id, dfrA14, fosA6, mdf*(*A*), *qnrB1, sul2* and *tet*(*A*) genes, conferring resistance to aminoglycosides, trimethoprim, fosfomycin, macrolides, quinolones and tetracyclines, respectively (Supplementary Table 2).

### Sequence Analyses and Synteny of IncI1-Iγ/ST3 Plasmids

Four IncI1-Iγ positive *E. coli* isolates were identified by a specific PCR among 51 3GCS-Ec isolated from frozen stools collected from 51 randomly chosen pets included in this study. An IncI1-Iγ/ST3 signature was recovered in one isolate (pEC345), the three others being assigned to ST7. The long-read sequencing approach was used to determine the full sequence of the previous IncI1-Iγ/ST3 plasmid and that of 2 *bla*_*CTX–M*–1_/IncI1-Iγ/ST3 plasmids, representative of the genomic diversity of our set of ESBL-Ec isolates, namely, pEC405 and pEC440 (Figure 1 and Table 2). To better characterize the plasmids circulating in Guadeloupe, three *bla*_*CTX–M*–1_/IncI1-Iγ/ST3 plasmids previously isolated in Guadeloupe from *E. coli* isolates associated with a human (pEC38), a bird (pEC1) and a rat (pEC7) were compared (Guyomard-Rabenirina et al., 2020). Plasmid pEC405 shared 98% of its sequence with pEC440 and pEC38, 94% with pEC1 and pEc7, 87% with pEC345. Nucleotide identity was > 99%. The plasmids can be divided into four functional modules consisting of antimicrobial resistance-encoding loci with plasmid replication, plasmid transfer, and plasmid maintenance functions. The *sul2* gene was present in both plasmids, *bla*_*CTX–M*–1_ being recovered in all ESBL-Ec (Table 2). No gene associated with resistance to heavy metal ions was identified. A type II toxin–antitoxin system consisting of YacA and YacB was present in all plasmids, and they encoded a colicin.

**TABLE 2:** Characteristics associated with IncI1-Iγ/ST3 plasmids from six *Escherichia coli* isolates collected in Guadeloupe.

Isolate name	Year of isolation	Host	Biological sample	MLST	Plasmid name	pMLST	Estimated plasmid size (bp)	Antibiotic resistance genes	Shufflon regions	Virulence determinants	Conjugation efficiency Mean*^a^* (SD)	Accession number
EC405	2019	Cat	Feces	ST328	pEC405	ST3	109,284	*bla_*CTX–M*–1_, tet*(*A*), *sul2*	CC′BB′AA′	Colicin	0.057 (0,008)	CP094200
EC440	2019	Dog	Feces	ST155	pEC440	ST3	108,832	*bla_*CTX–M*–1_, tet*(*A*), *sul2*	C′BB′CAA′	Colicin	0.081 (0.010)	CP094199
EC38	2013	Human	Blood	ST349	pEC38	ST3	108,442	*bla_*CTX–M*–1_, tet*(*A*), *sul2*	CC′BB′AA′	Colicin	0.083 (0.003)	CP053677
EC1	2013	Bird	Feces	ST196	pEC1	ST3	114,029	*aadA5*, *bla_*CTX–M*–1_, dfrA17*, *sul2*	B′BCC′AA′	Colicin	0.065 (0.017)	CP053560
EC7	2013	Rat	Feces	ST196	pEC7	ST3	114,029	*aadA5*, *bla_*CTX–M*–1_, dfrA17*, *sul2*	B′BCC′AA′	Colicin	0.031 (0.014)	CP053679
EC345	2019	Dog	Feces	Unknown	pEC345	ST3	109,373	*aadA1*, *bla*_*TEM*–1*B*_, *dfrA1*, *satA*, *sul2*	B′CA′	Colicin	Not done	CP094198

*^a^Conjugation experiments were performed in triplicate. All isolates produced an extended-spectrum β-lactamase (bla_CTX–M–1_), except for pEC345.*

**FIGURE 2:**
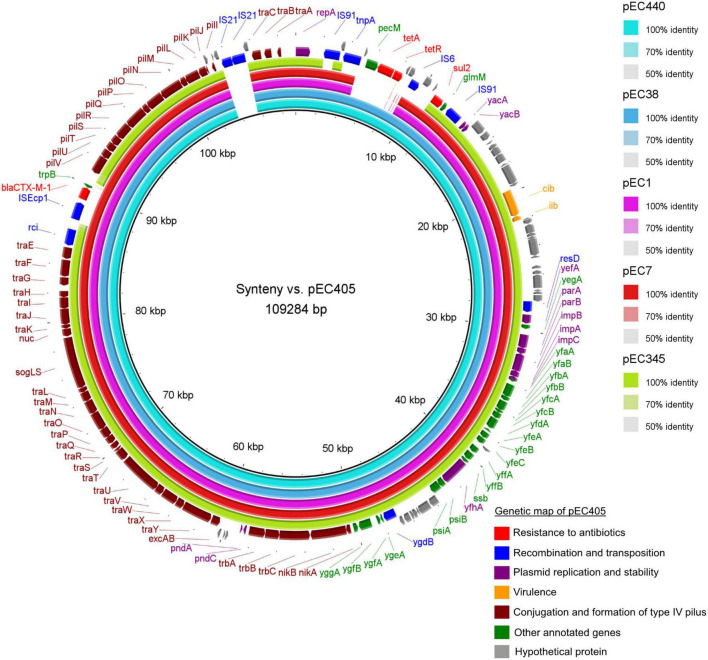
Genetic organization of accessory region carrying antibiotic resistance genes among IncI1-Iγ/ST3 plasmids from six *Escherichia coli* isolates collected in Guadeloupe (reference pEC405, 1 to 12,177 bp).

**FIGURE 3:**
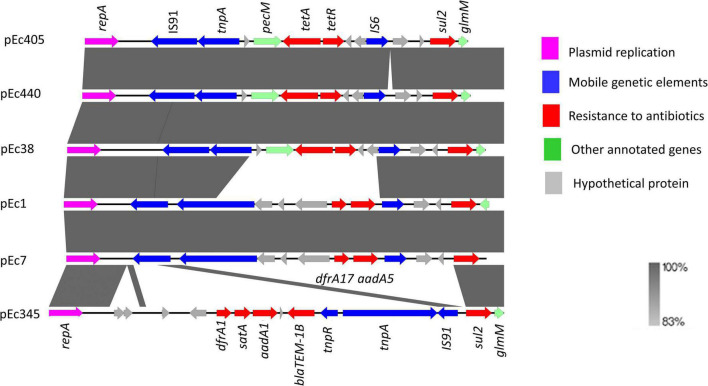
Syntenic analysis of 6 IncI1-Iγ/ST3 plasmids from six *Escherichia coli* isolates collected in Guadeloupe. All isolates produced an extended-spectrum β-lactamase (*bla*_*CTX–M*–1_) except for pEC345. The innermost black ring 1 represents the reference sequence of pEC405. The subsequent rings correspond to pairwise comparisons with pEC405: ring 2 represents pEC440; ring 3, pEC38; ring 4, pEC1; ring 5, pEc7; ring 6, pEC345. The last two rings represent a genetic map of pEC405.

Sequence alignment of the six IncI1-Iγ/ST3 signature plasmids showed the presence of two accessory regions containing antibiotic resistance genes (Figure 2). Smaller variations were observed at other locations on these plasmids. The first accessory region was delimited by *repA* (1–1,077 bp according to the reference plasmid pEC405) and by the *glmM* gene (11,875–12,177 bp) (Figure 3). The alignment was similar in ESBL-Ec plasmids from pets (pEC405 and pEC440) and from a human bloodstream infection (pEC38). The antimicrobial resistance genes *sul2* and *tet*(*A)* were present, as well as a non-functional truncated transposase belonging to the Tn3 family. A large section of the accessory module was different in pEC1 (bird) and pEC7 (rat) plasmids from previous plasmids (from 4,838 to 10,047 bp according to the reference plasmid pEC1). *aadA5*, *dfrA17*, and *sul2*, encoding resistance to streptomycin, trimethoprim, and sulfonamides, respectively, and a transposase belonging to the Tn3 family were present. This hypervariable region (from 2,401 to 14,462 bp according to the reference plasmid pEC345) was different in the plasmid associated with the 3GCS-*E. coli* (pEC345). A complete Tn3 transposon-containing *tnpA* encoding a Tn3 transposase, *tnpR* encoding a Tn3 resolvase, *dfrA1* coding for resistance to trimethoprim, *satA* conferring resistance to streptothricin, *aadA1* coding for resistance to streptomycin and spectinomycin, and *bla*_*TEM*–1–B_ encoding a β-lactamase were found. A *sul2* gene was detected outside the transposon as well as a truncated IS91 (Figure 3).

The *traABC* regulatory gene cluster was located at the distal end of the type IV pili locus region in IncI1-Iγ/ST3 plasmids. The shufflon consisted of several invertible DNA segments (Table 2) and was located at the proximal end of the type IV pili locus region. For 3GCR *E. coli*, *bla*_*CTX–M*–1_ was linked with ISEcp1 and inserted into the shufflon. It was delimited by the *rci* gene encoding a shufflon-specific recombinase and by the *pilV* gene encoding a membrane protein involved in fimbrial biogenesis. The plasmid pEC405 displayed an insertion of two copies of IS21 (from 103,656 to 105,457 pb) at proximity of the *traABC* cluster (Figure 2). This insertion was not present in the other plasmids.

The nucleotide sequence of our reference plasmid pEC405 was compared with the plasmid records stored in the PLSDB database by Mash with a maximal *P* of 0.1 and a distance threshold set at 0.01. A total of 55 IncI1-Iγ/ST3 plasmids (minimum 98,119 bp, maximum 123,053 bp, median 108,737 bp) were obtained (Supplementary Table 3), 52 (95%) displaying a *bla*_*CTX–M*–1_. The plasmids included in the hits were assigned to *E. coli* spp., except for one *Salmonella enterica*. They were isolated from a wide range of hosts, including animals [chicken (*n* = 21), pig (*n* = 8), turkey (*n* = 3), bird (*n* = 2), bovine (*n* = 2), dog (*n* = 1), duck (*n* = 1), rat (*n* = 1), wild boar (*n* = 1)], humans (*n* = 6) and the environment (*n* = 6) between 2002 and 2017, most having been extracted from samples collected in 2014 (*n* = 15). The isolates were mainly from Europe (*n* = 48), especially France (*n* = 10), North America (*n* = 4), Asia (*n* = 2) and Australia (*n* = 1). By computing pairwise distances with Mash with a distance cutoff of 0.00123693 (at least 950 of 1,000 shared hashes), we identified eight *E. coli* isolates harboring quasi-similar *bla*_*CTX–M*–1_/IncI1-Iγ/ST3 plasmids with the *tet*(*A)*/*sul2* region. According to the NCBI, they were found in four river samples from France, in fecal samples from two chickens and one pig from France and also in one human blood sample from Guadeloupe. The previous isolate was described above as pEC38. Syntenic analyses revealed that the previously described plasmid pEC38 shared 99% of its sequence with pEC405 while pEC440 was almost identical. Nucleotide identity was > 99.6%.

Conjugation efficiencies were substantially similar for pEC38, pEC405, pEC440, pEC1, and pEC7, between 3.1.10^–2^ and 8.34.10^–2^ (Table 2).

## Discussion

The overall prevalence of ESBL-E rectal carriage in pets (4.7%, 95% CI: 2.1–9.1) was moderate, in agreement with observations of fecal carriage among pigs (7.3%), beef cattle (14.7%) and humans (4.4%) in Guadeloupe (Gruel et al., 2021; Le Terrier et al., 2021). The prevalence of ESBL-E carriage was in the range observed worldwide (average, 6.9%) in a meta-analysis, the highest level being reported in dogs in Africa (16.5%) and in cats in Asia (16.8%) (Salgado-Caxito et al., 2021). The observed discrepancies may be due to the different populations investigated (e.g., healthy or ill individuals, geographic setting, species) or in sampling (clinical or non-clinical samples) and detection strategies.

A stay in a shelter was significantly associated with a higher prevalence of ESBL-E rectal carriage among cats, suggesting that refuges may be hotspots for their acquisition. To the best of our knowledge, this is the first time that shelters have been described as reservoirs of ESBL-E. It should be noted that domestic animals in shelters in Guadeloupe are often intended for adoption in mainland France, which would contribute to the geographic spread of ESBL-E. The ESBL-Ec isolates collected from pets living in the refuge were genetically almost identical (0–1 cgMLST allelic mismatches) and belonged to ST328, indicating spread of a single clone among pets. In view of the high ESBL-E prevalence among pets in the shelter, new hygiene and animal separation measures were implemented by veterinarians to prevent transmission. A second sampling campaign carried out in the refuge 1 year later on 15 pets that had not been sampled during the first campaign did not show the presence of ESBL-E rectal carriage (data not shown). Therefore, conditions such as overcrowding and lack of hygiene may have led to the infection. Chronic disease was significantly associated with a higher prevalence of ESBL-E rectal carriage among dogs, as previously described in humans (Hu et al., 2020).

Five STs were recovered, of which two were internationally successful clones (ST10 and ST155), both isolated from dogs. These two major clones have been described extensively in humans (Rafai et al., 2015), in domestic animals worldwide (Salgado-Caxito et al., 2021) and also in numerous other animal species, such as healthy poultry (Bortolaia et al., 2011) and migratory avian species (Mohsin et al., 2017). They were also isolated in two horses in Guadeloupe (Sadikalay et al., 2018), and ST10 was isolated from an iguana (Guyomard-Rabenirina et al., 2020). These cases highlight the potential role of companion animals in the transmission of ESBL-E to humans and vice versa. To the best of our knowledge, other genetic backgrounds such as ST328, ST953 and ST2739 have never or rarely been associated with pets. No *bla*_*CTX*–*M*–15_-producing *E. coli* ST131 was isolated in our study, even though the successful pandemic clone *E. coli* O25b:H4-ST131 is a serious concern worldwide (Coque et al., 2008; Rogers et al., 2011). Three ESBL-Ec ST155 isolates (EC435, EC440, and EC441) were separated by 0–1 SNPs. They were isolated from three dogs in the same household, indicating clonal transmission. Another isolate belonging to ST155 was separated from the three previous isolates by 70 SNPs and was isolated from another dog that had lived for 6 months in the same household as the other three dogs 18 months previously, indicating horizontal transmission. The only ESBL-producing *K. pneumoniae* was assigned to ST17, a successful human clone that has emerged as an important vehicle for worldwide dissemination of antibiotic-resistance genes (Huynh et al., 2020).

Most of ESBL-Ec isolated in this study (86%) carried *bla*_*CTX*–*M*–1_, in agreement with previous studies in Guadeloupe among healthy food-producing animals and wild fauna (Guyomard-Rabenirina et al., 2020; Gruel et al., 2021). *bla*_*CTX*–*M*–1_ is considered to be the most common ESBL in livestock and domestic animals (European Food Safety Authority and European Centre for Disease Prevention and Control, 2018). In our study, the *bla*_*CTX*–*M*–1_ gene from our ESBL-*E*c isolates was systematically associated with an IncI1-Iγ/ST3 plasmid. Two plasmids representative of the global genomic diversity of our set of ESBL-Ec isolates from pets were sequenced and found to be closely related to previously described *bla*_*CTX*–*M*–1_/IncI1-Iγ/ST3 plasmids found in three *E. coli* isolates from Guadeloupe associated with a human bloodstream infection and with feces from a rat and a bird (Guyomard-Rabenirina et al., 2020), although they were isolated 5 years apart. Two accessory modules carrying antimicrobial resistance genes were observed, in accordance with previous findings (Mo et al., 2020). In addition, shorter variations were observed at different positions of the plasmid, confirming that IncI1-Iγ can integrate accessory genes at different positions.

The *bla*_*CTX*–*M*–1_/IncI1-Iγ/ST3 plasmid is epidemiologically relevant, as it has been recovered in *E. coli* isolates from numerous animal and human sources in several countries in Europe, Asia and North America (Zurfluh et al., 2015; Baron et al., 2020; Moffat et al., 2020), indicating its significant role in the worldwide spread of *bla*_*CTX*–*M*–1_ genes (Carattoli et al., 2021). Furthermore, *bla*_*CTX*–*M*–1_/IncI1-Iγ/ST3 plasmids quasi similar to those found in *E. coli* from Guadeloupe (reference pEC405) were described in *E. coli* isolates from France recovered from four river samples and two chicken fecal samples, demonstrating its fitness, which contributes to its persistence in *E. coli* communities and its geographical spread. Its higher frequency in animals than in humans suggests an animal contribution to the CTX-M-1 reservoir in humans through the spread of this specific *bla*_*CTX*–*M*–1_/IncI1-Iγ/ST3 plasmid. To increase knowledge on its emergence, we investigated the prevalence of IncI1-Iγ/ST3 plasmids in 3GCS *E. coli* in domestic animals. Although it is rare (only one isolate), its presence should be considered in the perspective of the ubiquitous character of *E. coli*. This group of plasmids is present, suggesting that they have acquired resistance to 3GC by insertion of *bla*_*CTX*–*M*–1_ into existing plasmids in animals rather than by wide spread of previously uncommon plasmids. The use of antibiotics in animals has probably favored their spread.

Although plasmids are abundant in bacterial populations, these genetic elements are generally responsible for a decrease in fitness due to physiological alterations in their bacterial hosts (Alonso-Del Valle et al., 2021). The only potential advantages associated with acquisition of *bla*_*CTX*–*M*–1_/IncI1-Iγ/ST3 are associated with the presence of antibiotic resistance genes. In an *in vivo* model in chickens, competitive exclusion of *E. coli* carrying an *bla*_*CTX–M*–1_/IncI1-Iγ/ST7 could decrease the levels of bacteria carrying this plasmid through differences in growth rate compared to resident bacteria already present in the microbiome (Fischer et al., 2019). Different strategies can be proposed to explain the persistence of plasmids in bacterial populations over long periods in the absence of direct selection pressure. *In vitro* studies have shown that IncI1 plasmids harboring *bla*_*CTX*–*M*–1_ show no or negligible fitness loss in ESBL-Ec strains and that these plasmids can persist for long periods in the absence of antimicrobial selection (Fischer et al., 2014). All these plasmids display a toxin/antitoxin system that is beneficial for stable inheritance of the plasmids and encode a colicin that represent a selective advantage (Majeed et al., 2011), as it can successfully compete with related bacteria in the gut without colicin production. In addition, *in vivo* and *in vitro* studies demonstrate that horizontal transfer of the ESBL gene by conjugation can outweigh competition by growth, in the absence of antibiotics, resulting in expansion and persistence of IncI1 plasmids carrying *bl*a_*CTX–M*–1_, in agreement with the high conjugation efficiency of our IncI1-Iγ/ST3 plasmids (Fischer et al., 2019). This is worrisome, as, if this hypothesis is confirmed, a reduction in antibiotic use is probably insufficient to reverse the trend, and new strategies will be required to inhibit conjugation (Lopatkin et al., 2017).

In conclusion, we found a moderate prevalence of ESBL-E fecal carriage in healthy dogs and cats in Guadeloupe (7.6%). Well-conserved *bla*_*CTX*–*M*–1_/IncI1-Iγ/ST3 plasmids are spread widely among domestic animals and humans in Guadeloupe. The findings stress the importance of basic hygiene measures for owners in close proximity to companion animals.

## Data Availability Statement

The datasets presented in this study can be found in online repositories. The names of the repository/repositories and accession number(s) can be found below: NCBI, PRJNA798606, PRJNA600948, and PRJNA798907.

## Ethics Statement

The animal study was reviewed and approved by Committee for Ethics in animal experiments of the French West Indies and Guyana. Written informed consent was obtained from the owners for the participation of their animals in this study.

## Author Contributions

SG-R, J-CB, SF, and SB conceived and designed the study. GG, SG-R, and SF collected biological samples, isolates, and epidemiological data. GG, DC, GA, and SB analyzed the data. BT performed the statistical analyses. GG, DC, and SB wrote the manuscript. All authors critically revised the manuscript and read and approved the final manuscript.

## Conflict of Interest

The authors declare that the research was conducted in the absence of any commercial or financial relationships that could be construed as a potential conflict of interest.

## Publisher’s Note

All claims expressed in this article are solely those of the authors and do not necessarily represent those of their affiliated organizations, or those of the publisher, the editors and the reviewers. Any product that may be evaluated in this article, or claim that may be made by its manufacturer, is not guaranteed or endorsed by the publisher.

## Footnotes

1
https://github.com/tseemann/mlst

2
https://github.com/tseemann/abricate

3
https://nanoporetech.com/

## Supplementary Material

Supplementary Table 1 Comparison of differential SNPs detected in 13 extended-spectrum β-lactamase-producing *Escherichia coli* isolates collected in Guadeloupe (French West Indies).

Supplementary Table 2 Molecular characteristics of 13 extended-spectrum β-lactamase-producing *Escherichia coli* isolates from pets in Guadeloupe (French West Indies).

Supplementary Table 3 Metadata on IncI1-Iγ/ST3 plasmids in the PLSDB database that share at least 800 of 1000 hashes with pEC405.
